# Bacterial Diversity of Root Nodule and Rhizosphere Soil Samples of Green Soybean (Edamame) in Japan

**DOI:** 10.1128/mra.01114-21

**Published:** 2022-02-03

**Authors:** Daisuke Fukuda, Naoko Ohnuki, Takashi Ohnuki

**Affiliations:** a Medical Affairs, Merck Research Laboratories, MSD K.K., Chiyoda-ku, Tokyo, Japan; b Ebinamame, Ebina-shi, Kanagawa, Japan; University of Southern California

## Abstract

We analyzed the bacterial diversity of root nodule and rhizosphere soil samples of edamame collected in Ebina, Japan. The major population identified from the nodules belonged to the genus *Bradyrhizobium*, and the rhizosphere soil in the open field has a higher abundance of the *Rhizobiales* order bacteria than that in the greenhouse.

## ANNOUNCEMENT

Soybean rhizosphere soils and root nodules contain a wide range of microbial diversity of both rhizobial and nonrhizobial endophytes (1, 2), and *Bradyrhizobium* is known to be dominantly present in the root nodules of various soybean varieties (3, 4). Edamame (Glycine max [L.] Merrill), also referred to as green soybean or vegetable soybean, has long been an accepted part of Japanese food culture. We assessed the bacterial communities of edamame root nodules and rhizosphere soils from greenhouse and open-field plants using high-throughput 16S rRNA gene amplicon sequencing.

**FIG 1:**
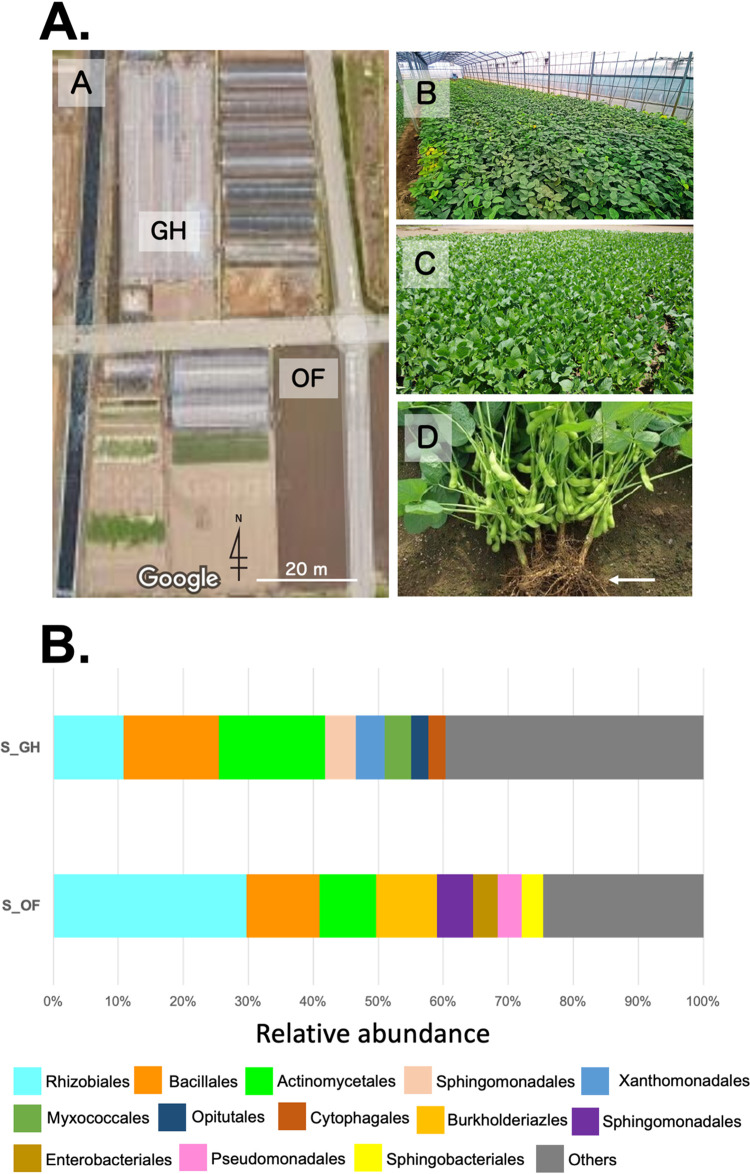
(A) Overview of the sampling locations in the edamame farm (Ebina, Kanagawa, Japan) (image A). Both the nodule and soil samples were collected from greenhouse (GH) and open-field (OF) farms. The image was obtained from Google Maps. The green soybeans were routinely grown in the greenhouse (image B) and open field (image C). The root nodule and soil samples were collected from the rhizosphere zone of edamame indicated by the arrow (image D). (B) Bar chart representing the taxonomic composition of the microbiota of rhizosphere soil samples S_GH (greenhouse) and S_OF (open field) based on 16S rRNA gene amplicon sequence analysis. Relative abundances of taxa are shown at the order level.

Nodules and soils for analysis were sampled at a depth of 0 to 20 cm in edamame farms in Ebina, Kanagawa, Japan (GPS location N35.42, E139.39). The sampling locations in the edamame farm are described in Fig. 1A. The seeds were sown in February 2021. The plants were grown in a greenhouse and in an open field. After 3 months of cultivation, rhizosphere soils and nodule samples were collected at the full-bloom stage in May 2021. The samples are summarized in Table 1.

Three green soybean plants were randomly selected from of the greenhouse and open-field plants. To isolate nodules, plants were uprooted, and the nodules were transferred into test tubes. Then, the nodules were washed under running tap water, surface-sterilized in a 3% sodium hypochlorite solution for 3 min, and rinsed three times in sterilized distilled water. To collect rhizosphere soils, the soil adhering to the roots was brushed off with a toothbrush. Total DNA was extracted from 0.25 g of freeze-dried rhizosphere soil and nodulation samples (three to four nodules in each sample) using an MPure bacterial DNA extraction kit (MP Bio) according to the manufacturer’s protocol. The QuantiFluor double-stranded DNA (dsDNA) system (Promega) was used to check the extracted DNA concentration. The V4 to V5 regions of the bacterial 16S rRNA gene were amplified by two-step PCR using the primer sets and method previously reported (5). Paired-end (2 × 300 bp) sequencing was conducted with the Illumina MiSeq platform using the MiSeq reagent kit v3 (Illumina). The raw data obtained were assembled with high-quality scores (average score, >20). The paired-end demultiplexed sequences were imported using QIIME2 v2020.6 (6), and the DADA2 plugin was used to denoise the sequences and to remove chimeric sequences (7). Default parameters were used for all of the software contained in the QIIME2 environment. Greengenes v13.8 was utilized to conduct operational taxonomic unit (OTU) clustering analysis at 97% identity (8). The chloroplast and mitochondrial OTUs were removed for further analysis.

We obtained 497,857 raw reads from 8 nodule and soil samples (Table 1) (62,232 reads/sample on average), 346,779 (69.7%) of which remained after quality filtering.

**TABLE 1:** Characteristics of samples and sequencing data

Sample no.	Sample name	Sample type	Cultivation location	SRA accession no.	No. of raw sequence reads	No. of filtered
						reads (nonchimera)
1	N_GH1	Nodule	Greenhouse	DRX311395	53,252	41,426
2	N_GH2	Nodule	Greenhouse	DRX311396	62,335	48,337
3	N_GH3	Nodule	Greenhouse	DRX311397	62,956	46,923
4	N_OF1	Nodule	Open field	DRX311398	55,963	43,145
5	N_OF2	Nodule	Open field	DRX311399	65,174	51,318
6	N_OF3	Nodule	Open field	DRX311400	60,240	47,050
7	S_GH	Soil	Greenhouse	DRX311401	67,870	31,924
8	S_OF	Soil	Open field	DRX311402	70,067	36,656

A total of 1,187 unique OTUs were detected in all samples, with the nodule samples composed of only 1 to 2 OTUs and the soil samples containing 596 to 640 OTUs. Bacterial taxonomic analysis showed that all nodule samples of green soybean from both a greenhouse and open field were highly dominated by the *Bradyrhizobium* genus (>99.9%; data not shown in Fig. 1B due to high abundance). In the bacterial order, the microbiota of rhizosphere soils from a greenhouse and an open field shared *Rhizobiales*, *Actinomycetales*, *Bacillales*, and *Sphingomonadales* (Fig. 1B). *Rhizobiales* was most highly present in sample S_OF (rhizosphere soil from open field), at 29.7%. Sample S_GH (rhizosphere soil from greenhouse) exhibited a predominance of *Actinomycetales* and *Bacillales*, followed by *Rhizobiales* (relative abundance of 16.3%, 14.6%, and 10.9%, respectively).

We will further investigate the contribution of differences in rhizospheric microbial compositions due to cultivation conditions to the quality of edamame.

### Data availability.

The 16S rRNA gene amplicon data sets have been deposited at DDBJ/ENA/GenBank under the accession number PRJDB12426 and can be accessed in the SRA under the accession numbers DRX311395 (N_GH1), DRX311396 (N_GH2), DRX311397 (N_GH3), DRX311398 (N_OF1), DRX311399 (N_OF2), DRX311400 (N_OF3), DRX311401 (S_GH), and DRX311402 (S_OF).
